# Added value of CE-CT radiomics to predict high Ki-67 expression in hepatocellular carcinoma

**DOI:** 10.1186/s12880-023-01069-4

**Published:** 2023-09-22

**Authors:** Yu-meng Zhao, Shuang-shuang Xie, Jian Wang, Ya-min Zhang, Wen-Cui Li, Zhao-Xiang Ye, Wen Shen

**Affiliations:** 1 Medical School of Nankai University, No. 94, Weijin Road, Nankai District, Tianjin, China; 2 Department of Radiology, Tianjin First Center Hospital, Tianjin Institute of imaging medicine, School of Medicine, Nankai University, Nankai District, No. 24 Fukang Road, Tianjin, China; 3 Department of Hepatobiliary Surgery, Tianjin First Central Hospital, School of Medicine, Nankai University, Nankai District, No. 24 Fukang Road, Tianjin, China; 4 Department of Radiology, Tianjin Medical University Cancer Institute and Hospital, National Clinical Research Center for Cancer, Tianjin, 300060 China

**Keywords:** Hepatocellular carcinoma, Ki-67 expression, Contrast-enhanced computed tomography, Radiomics

## Abstract

**Background:**

This study aimed to develop a computed tomography (CT) model to predict Ki-67 expression in hepatocellular carcinoma (HCC) and to examine the added value of radiomics to clinico-radiological features.

**Methods:**

A total of 208 patients (training set, *n* = 120; internal test set, *n* = 51; external validation set, *n* = 37) with pathologically confirmed HCC who underwent contrast-enhanced CT (CE-CT) within 1 month before surgery were retrospectively included from January 2014 to September 2021. Radiomics features were extracted and selected from three phases of CE-CT images, least absolute shrinkage and selection operator regression (LASSO) was used to select features, and the rad-score was calculated. CE-CT imaging and clinical features were selected using univariate and multivariate analyses, respectively. Three prediction models, including clinic-radiologic (CR) model, rad-score (R) model, and clinic-radiologic-radiomic (CRR) model, were developed and validated using logistic regression analysis. The performance of different models for predicting Ki-67 expression was evaluated using the area under the receiver operating characteristic curve (AUROC) and decision curve analysis (DCA).

**Results:**

HCCs with high Ki-67 expression were more likely to have high serum α-fetoprotein levels (*P* = 0.041, odds ratio [OR] 2.54, 95% confidence interval [CI]: 1.04–6.21), non-rim arterial phase hyperenhancement (*P* = 0.001, OR 15.13, 95% CI 2.87–79.76), portal vein tumor thrombus (*P* = 0.035, OR 3.19, 95% CI: 1.08–9.37), and two-trait predictor of venous invasion (*P* = 0.026, OR 14.04, 95% CI: 1.39–144.32). The CR model achieved relatively good and stable performance compared with the R model (AUC, 0.805 [95% CI: 0.683–0.926] vs. 0.678 [95% CI: 0.536–0.839], *P* = 0.211; and 0.805 [95% CI: 0.657–0.953] vs. 0.667 [95% CI: 0.495–0.839], *P* = 0.135) in the internal and external validation sets. After combining the CR model with the R model, the AUC of the CRR model increased to 0.903 (95% CI: 0.849–0.956) in the training set, which was significantly higher than that of the CR model (*P* = 0.0148). However, no significant differences were found between the CRR and CR models in the internal and external validation sets (*P* = 0.264 and *P* = 0.084, respectively).

**Conclusions:**

Preoperative models based on clinical and CE-CT imaging features can be used to predict HCC with high Ki-67 expression accurately. However, radiomics cannot provide added value.

**Supplementary Information:**

The online version contains supplementary material available at 10.1186/s12880-023-01069-4.

## Background

Hepatocellular carcinoma (HCC) is the most common type of primary liver carcinoma in adults and the third leading cause of cancer-related deaths worldwide [1]^.^ Despite advances in surgical resection, a high rate of recurrence and metastasis remains, leading to a poor prognosis of HCC after surgical resection.

As a nuclear antigen, Ki-67 is highly expressed in malignant cells but cannot be detected in normal cells, and its proliferation index reflects the station of tumor proliferation activity and has a strong relationship with tumor grade [2]. Previous studies have shown that patients with HCC with high Ki-67 expression have a significantly poor prognosis in terms of recurrence rates, overall survival (OS), disease-free survival (DFS), and relapse-free survival (RFS) [2, 3]. In addition, Ki**-**67-targeted strategies for renal carcinoma have been shown to be effective in killing renal carcinoma cells and prolonging patients’ prognosis [4]. Therefore, Ki-67 has become a promising target for other solid cancer therapies such as HCC [4, 5]. To date, the gold standard for the diagnosis of Ki-67 relies on surgical specimens that involve a substantial time delay for patients with HCC. If Ki-67 can be predicted before surgery, patients with HCC may receive more appropriate treatment procedures (such as targeted therapies alone or in combination with locoregional therapy). Therefore, it is important to forecast the Ki-67 status using a non-invasive method before surgery.

With great advances in artificial intelligence and computing equipment, radiomics has flourished. Radiomics quantifies and characterizes the biological characteristics of tumors through a large number of quantitative features that are transformed from visual images. It is expected to achieve non-invasive, comprehensive, and dynamic quantification of the temporal and spatial heterogeneity of lesions. Thus, radiomics has important clinical value for the accurate diagnosis and treatment of diseases and prognosis prediction. Radiomics has been widely studied for the diagnosis and treatment of diseases [6, 7]. Radiomics features based on magnetic resonance imaging (MRI) images combined with laboratory factors and/or imaging features to develop HCC Ki-67 expression prediction models are well recognized [5, 8, 9]. Contrast-enhanced computed tomography (CE-CT) is widely used in clinics for the detection and diagnosis of HCC and is relatively inexpensive and rapid. However, previous radiomic studies based on CE-CT images only used the arterial and portal venous phases and ignored the important value of the delayed phase [10], which cannot reflect whole tumor characteristics. In addition, they did not include traditional imaging features, which are important in daily work. Finally, these studies did not include an external validation set to verify further the model’s stability and generalizability [11, 12]. Therefore, whether radiomics based on CE-CT analysis can add ancillary value to predict Ki-67 expression remains unclear, and the stability of radiomics needs to be further explored.

Therefore, this study aimed to develop, test, and validate a clinic-radiologic (CR) model based on CE-CT imaging features, a rad-score model based on three phases of CE-CT imaging radiomics features, and a combined clinic-radiologic-radiomic (CRR) model to predict Ki-67 expression in HCC preoperatively, then compare the stability of the CR and rad-score model, and investigate the added value of radiomics features.

## Methods

### Study population

**Fig. 1:**
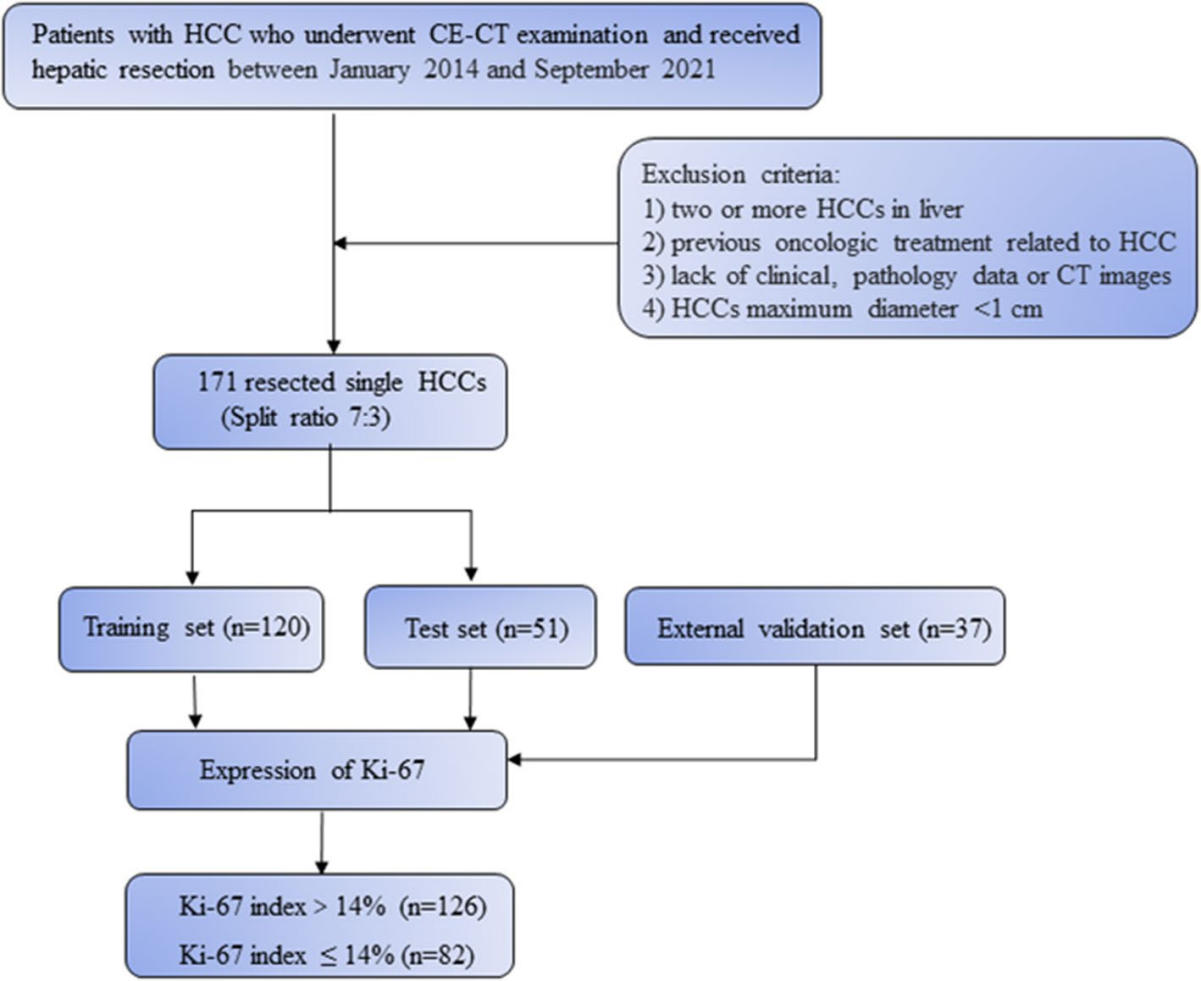
Flowchart of study inclusion. HCC, hepatocellular carcinoma

This retrospective study was approved by the institutional review boards of the participating centers, and the informed consent from patients was waived off. Between January 2014 and September 2021, 171 consecutive patients with HCC who underwent preoperative CE-CT examination and surgery in center 1 were enrolled. All enrolled patients were randomly allocated to the training (*n* = 120) and internal test sets (*n* = 51) in a 7:3 ratio. A total of 37 patients with HCC from center 2 were enrolled as an external validation set. The inclusion criteria were as follows: 1) patients with pathologically proven solitary HCC; 2) patients who underwent abdominal CE-CT, including arterial phase (AP), portal venous phase (PP), and delayed phase (DP), within 1 month before surgery (hepatectomy or liver transplantation); 3) patients who had a post-surgery immuno-oncologic characteristic diagnosis of HCC with a definite Ki-67 status; and 4) patients who had not undergone any oncologic treatment before surgery (liver transplantation, hepatectomy, chemotherapy, radiotherapy, or systemic immunotherapy). The workflow of this study is shown in Fig. 1.

### Preoperative clinical and histopathological data

The following baseline data were obtained from the medical records: age, sex, tumor number and size, cirrhosis, liver disease etiology, serum liver function indexes, and serum tumor markers.

Ki-67 expression was evaluated by calculating the percentage of positively stained cells (cell nuclei stained brown-yellow). Immunoreactivity in > 14% of tumor cells was classified as high Ki**-**67 expression and ≤ 14% as low Ki-67 expression [4, 9]. Pathological data were obtained from the pathology departments of each center. All surgical specimens were reviewed by two pathologists from the two centers. In case of disagreement, a consensus was reached through discussion.

### Preoperative CT image features

#### CT examination

CT scans of the two centers were made using Toshiba Aquilion 64-layer CT, GE Revolution CT, GE Discovery 64-layer Screw CT, GE Lightspeed 16-layer Spiral CT, Siemens Dual Source CT, and Siemens 128-layer CT devices. The patient was placed in a supine position, and the scan range was from the top of the diaphragm to the lower stage of both kidneys. Scanning parameters: tube voltage 120 kV, automatic adjustment of tube current, scanning layer thickness and layer spacing are 5 mm, reconstruction layer thickness is 1.25 mm or 1.2 mm, rotation time 0.5 s/r, field of view 350–400 mm × 3 50–400 mm, matrix 256 × 256 or 512 × 512, Siemens dual-source CT and Siemens 128–layer CT pitch is 0.900:1, other CT scanning equipment pitch is 0.984:1. All patients were given elbow intravenous injection of contrast medium idohexol (containing iodine 350 mg/mL, Shanghai General Electric Pharmaceutical), injection dose 1.0–1.5 mL/kg body mass, and flow rate 3.5–4.0 mL/s. Using contrast agent tracing trigger technology, the trigger point was set at the beginning of the abdominal aorta, and the scanning scan began after injection of contrast medium for 15 s. The trigger threshold was 120 HU, the intra-abdominal aorta CT value reached 120 HU when the arterial phase was scanned, and the portal vein and delayed phase scans were performed after 30 s and 300 s, respectively.

#### Image analysis

Analysis of all LI-RADS v2018 major (except for those related to growth since these data were unavailable in the original registry) and ancillary features and some other important imaging features based on CE-CT were performed retrospectively by two independent radiologists (Xie with 10 years and Zhao with 5 years of experience) who were blinded to related clinical and pathological information. The radiologists assessed the following CE-CT features for each patient (Supplement Fig. 1): (a) maximum tumor length (L-max ≤ 5 cm; L-max > 5 cm; (b) non-rim arterial phase hyperenhancement (APHE); (c) non-peripheral washout; (d) tumor capsule; (e) capsule enhancement; (f) corona enhancement; (g) nodule in nodule sign; (h) mosaic architecture; (i) scar sign; (j) tumor rupture; (k) intratumor necrosis; (l) portal vein tumor thrombus (PVTT); (m) two-trait predictor of venous invasion (TTPVI) (consisting of “internal arteries” and “hypodense halos”); and (n) peritumoral satellite. The reproducibility of all intra- and interobserver features was assessed using Cohen’s Kappa. Features with Kappa values greater than 0.7 were considered reproducible and included in the following feature selection.

#### Image segmentation and radiomic analysis

Image segmentation was performed in the open-source 3D-Slicer 4.10.2 software (https://download.slicer.org/) and was based on the expert consensus on CT and MRI labeling of liver visceral focal lesions (2020 edition). The three-dimensional volume of interest (VOI) containing the tumor mass was manually outlined layer by layer by two radiologists (with 5 and 10 years of experience in abdominal imaging diagnosis, respectively) on arterial, portal venous, and delayed phase.

Radiomic feature extraction: before the feature extraction, standardized preprocessing (including voxel size resampling and gray-level discretization) was conducted. Radiomics packets in 3D-Slicer software were used to extract the features of the outlined VOI, and all images were processed standard before extraction. First, to reduce the influence of image size on the result, all images were resampled to a uniform scale, the image grayscale was uniformized, the Gauss Laplace filter and wavelet filter were used for filtering, and 1,037 features were obtained in the arterial, portal venous, and delayed phases. The inter-observer reproducibility of all radiomic features extracted from the VOI was analyzed, and features with an inter-observer intra-class correlation coefficient (ICC) ≥ 0.8 were included for subsequent radiomic analysis.

Using the "StandardScaler, " "Levene," and "LassoCV" packages in R Studio to achieve data normalization, one-factor analysis, and decile-fold cross-validation of minimum absolute convergence and selection operator (LASSO) regression by optimizing the regression parameters (λ), most of the eigenvalues were reduced to zero, select the remaining non-zero coefficient features to obtain omics features that are highly correlated with Ki-67 expression. The radiomics features obtained from the training set were used to calculate the radiomics score (rad-score) of each patient.

### Model development

A CR model was developed based on clinical and radiological features selected from univariate and multivariate logistic regression analyses. Similarly, a combined model, the CRR model, was developed based on clinical, radiological, and radiomic features selected from univariate and multivariate logistic regression analyses. The CRR model was used to test whether the radiomic signature and clinical features were complementary for the prediction of Ki-67 expression. The diagnostic ability of the two models was evaluated based on the area under the receiver operating characteristic (ROC) curve (AUC value), and the DeLong test was used to select the best model. Decision curve analysis (DCA) was performed by quantifying the net benefit at all threshold probabilities to determine the clinical utility of the model.

**Fig. 2:**
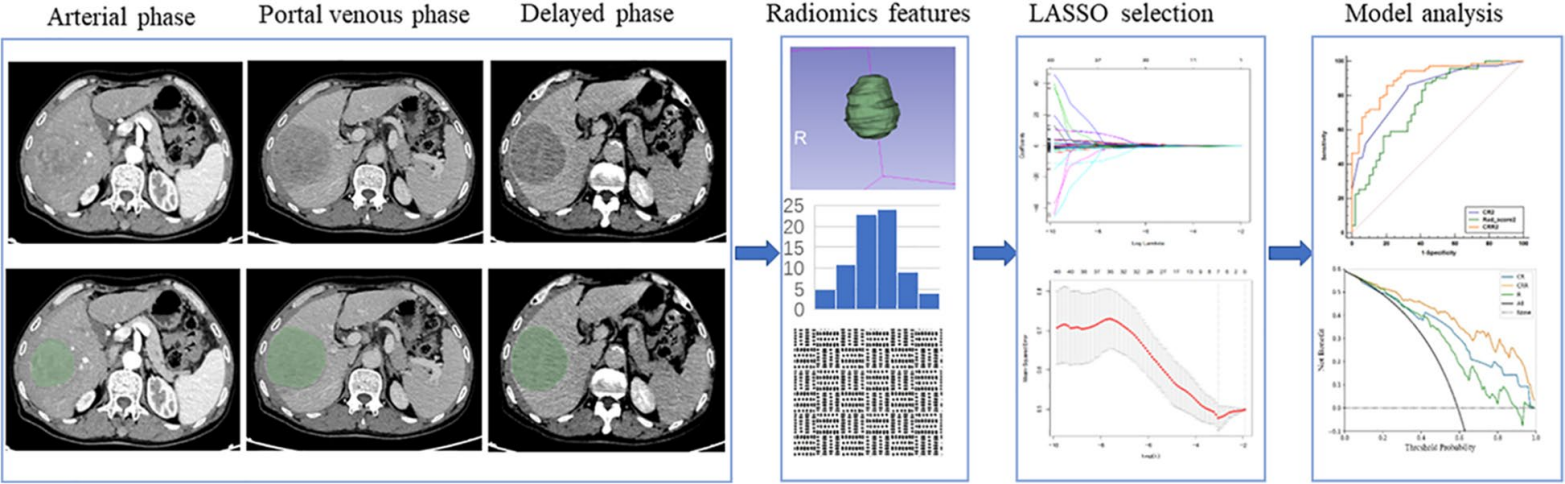
Flowchart of tumor segmentation, feature extraction, and model building. Manual segmentation was performed in the multi-phase images, and radiomics features were extracted. Then, LASSO was used for radiomics feature selection and finally, a model was establishment. Receiver operating characteristic (ROC) curve and the DCA curve for predicting Ki-67 status were then developed. LASSO, least absolute shrinkage and selection operator; DCA, Decision curve analysis

A flowchart of the image segmentation, radiomics feature extraction, and model development is shown in Fig. 2.

### Statistical analysis

Data analysis was performed to select features using the Statistical Package for the Social Sciences v26 software (IBM, Armonk, NY, USA) and R software (version 4.1.2). The continuous variables were described as medians and interquartile ranges, and categorical variables were described as frequencies and percentages. The statistical differences (between the training and validation sets and between high Ki-67 expression and low Ki-67 expression groups in the training and validation sets) of continuous variables were compared using either the t-test or Mann–Whitney U test, and categorical variables were compared using either the χ^2^ test or Fisher’s exact test. Interobserver variability was determined using Cohen’s Kappa coefficient for categorical variables. ROC curves were created using the MedCalc software (version 19.6), and the DeLong test was used to compare the differences in AUC values between the groups.

All statistical tests were two-sided, and a *P*-value < 0.05 was considered statistically significant.

## Results

### Baseline characteristics

A total of 208 patients were recruited; 171 patients from center 1 were classified into a training set (*n* = 120, 49 for low Ki-67 expression, 71 for high Ki-67 expression; 105 men and 15 women) and an internal test set (*n* = 51, 21 for low Ki-67 expression, 30 for high Ki-67 expression; 41 men and 10 women). Moreover, 37 patients from center 2 (27 men and 10 women; 12 with low Ki-67 expression and 25 with high Ki-67 expression) were included in the validation set. There were no significant differences in the demographic and laboratory features among the training, test, and externally validated patients (Table 1). According to the Kappa value (> 0.7), 13 CT imaging features were selected (Supplement Table 1). Comparisons of CE-CT imaging features between the high and low Ki-67 expression groups of each set are shown in Table 2.

**Table 1:** Comparison of Ki67 status and characteristics in both training and test HCC patients

Characteristic	Model derivation and verification		Model application	*P*
	Training set (*n* = 120)	Internal test set (*n* = 51)	External validation set (*n* = 37)	
Sex				0.255
Male	105	41	27	
Female	15	10	10	
Age	56.55 ± 9.53	56.14 ± 10.82	58.32 ± 9.88	0.804
Etiology				0.606
HBV/HCV	107	47	19	
None or other	13	4	18	
Serum AFP				0.995
Normal	47	20	8	
Abnormal	73	31	29	
Total bilirubin (nmol/L)	17.15(11.43–32.04)	17.11(11.71–31.15)	15.8(12.0–19.75)	0.828
ALB(g/l)	37.85(32.00–42.10)	37.80(31.80–42.10)	43.5(39.55–45.05)	0.412
CA199(ng/ml)	19.89(10.44–45.11)	19.90(13.42–46.1)	16.84(10.59–26.21)	0.446
CEA (ng/ml)	2.53(1.78–3.69)	2.63(1.89–3.65)	2.11(1.61–4.13)	0.056

*HBV* hepatitis B varus, *HCV* hepatitis C varus, *AFP* α-fetoprotein, *ALB* albumin, *CA-199* carbohydrate antigen19-9, *CEA* carcino embryonie antigen

**Table 2:** CE-CT imaging features between high Ki-67 expression and low Ki-67 expression groups of training set, internal test set and external validation set

Characteristic		Training set (*n* = 120)				Internal test set (*n* = 51)				External validation set (*n* = 37)			
		Low Ki67(*n* = 49)	High Ki67(*n* = 71)	n	*P* value	Low Ki67(*n* = 21)	High Ki67(*n* = 30)	n	*P* value	Low Ki67(*n* = 12)	High Ki67(*n* = 25)	n	*P* value
Size	> 5 cm	19(29.7)	45(70.3)	64	0.01	13(48.1)	14(51.9)	27	0.394	5(26.3)	14(73.7)	19	0.495
	≤ 5 cm	30(53.6)	26(46.4)	56		8(33.3)	16(66.7)	24		7(38.9)	11(61.1)	18	
Non-rim APHE	Yes	33(32.7)	68(67.3)	101	< 0.001	13(34.2)	25(65.8)	38	<0.109	6(22.2)	21(77.8)	27	<0.109
	No	16(84.2)	3(15.8)	19		8(61.5)	5(38.5)	13		6(60.0)	4(40.0)	10	
Non-peripheral washout	Yes	38(35.8)	68(64.2)	106	0.003	18(42.9)	24(57.1)	42	0.72	8(33.3)	16(66.7)	24	0.874
	No	11(78.6)	3(21.4)	14		3(33.3)	6(66.7)	9		4(30.8)	9(69.2)	13	
Corona enhancement	Yes	7(35.0)	13(65.0)	20	0.625	4(36.4)	7(63.6)	11	0.746	0(0.0)	1(100.0)	1	0.482
	No	42(42.0)	58(58.0)	100		17(42.5)	23(57.5)	40		12(33.3)	24(66.7)	36	
Nodule-in-nodule architecture	Yes	7(58.3)	5(41.7)	12	0.161	1(33.3)	2(66.7)	3	0.776	2(22.2)	7(77.8)	9	0.687
	No	42(38.9)	66(61.1)	108		20(41.7)	28(58.3)	48		10(35.7)	18(64.3)	28	
Mosaic architecture	Yes	33(36.3)	58(63.7)	91	0.057	17(47.2)	19(52.8)	36	0.221	2(18.2)	9(81.8)	11	0.279
	No	16(55.2)	13(44.8)	29		4(26.7)	11(73.3)	15		10(38.5)	16(61.5)	26	
Scar sign	Yes	2(28.6)	5 (71.4)	7	0.398	1(20.0)	4(80.0)	5	0.391	3(27.3)	8(72.7)	11	0.722
	No	47(41.6)	66(58.4)	113		20(43.5)	26(56.5)	46		9(34.6)	17(65.4)	26	
Tumor rupture	Yes	1(33.3)	2(66.7)	3	0.637	0(0.0)	2(100.0)	2	0.506	0(0.0)	0(0.0)		--
	No	48(41.0)	69(59.0)	117		21(42.9)	28(57.1)	49		12(32.4)	25(67.6)	37	
Intra-tumoral necrosis	Yes	21(32.8)	43(67.2)	64	0.065	15(42.9)	20(57.1)	35	0.768	4(28.6)	10(71.4)	14	0.735
	No	28(50.0)	28(50.0)	56		6(37.5)	10(62.5)	16		8(34.8)	15(65.2)	23	
TTPVI	Yes	1(5.0)	19(95.0)	20	< 0.001	6(28.6)	15(71.4)	21	0.156	0(0.0)	4(100.0)	4	0.142
	No	48(48.0)	52(52.0)	100		15(50.0)	15(50.0)	30		12(36.4)	21(63.3)	33	
PVTT	Yes	8(20.5)	31(79.5)	39	0.003	7(46.7)	8(53.3)	15	0.757	0(0.0)	3(100.0)	3	0.537
	No	41(50.6)	40(49.4)	81		14(38.9)	22(61.1)	36		12(35.3)	22(64.7)	34	
peritumoral satellite	Yes	6(21.4)	22(78.6)	36	0.027	5(41.7)	7(58.3)	12	0.969	0(0.0)	0(0.0)		--
	No	43(46.7)	49(53.3	135		16(41.0)	23(59.0)	39		12(32.4)	25(67.6)	37	

### Radiomic feature selection and performance of radiomic signature

A total of 3,111 intra-tumoral features were extracted from the AP, PP, and DP images. After ICC, data normalization and LASSO were described in the Methods section. Seven radiomic features were selected for the construction of the radiomic signature. Among these features, four, two, and three features from AP, PP, and DP, respectively, and four were first-order features, two were gray level size zone matrix (GLSZM), and one was a gray level co-occurrence matrix (GLCM); feature names and their weights are shown in Supplement Fig. 2. The AUC value of the radiomic signature was 0.728 (95% confidence interval [CI]: 0.659–0.796) in the training set and 0.711 (95% CI: 0.640–0.781) in the test set and external validation set (Table 3).

**Table 3:** Factors significantly associated with Ki-67 expression

Variables	Multivariate analysis1		Multivariate analysis2	
	OR(95%CI)	*P*	OR(95%CI)	*P*
AFP(ng/ml)	2.54(1.04–6.21)	0.041	3.17(1.11–9.01)	0.031
APHE(absent)	15.13(2.87–79.76)	0.001	20.63(3.71–114.68)	0.001
PVTT(absent)	3.19(1.08–9.37)	0.035	2.69(0.793–9.11)	0.043
TTPVI(absent)	14.04(1.39–144.32)	0.026	2.92(1.72–4.96)	0.046
Rad-score				< 0.001

Multivariable analysis 1: multivariate analysis with clinic-radiologic(CR) model variables. Multivariable analysis 2: multivariate analysis with clinic-radiologic-radiomic(CRR) model variables and radiomic signature, *OR* odds ratio, *CI* confidence interval, *AFP* alpha-fetoprotein, *APHE* arterial phase hyperenhancement, *PVTT* portal venous tumor, *TTPVI* two-trait predictor of venous invasion

### Feature selection and predictive model development for Ki-67 expression

**Fig. 3:**
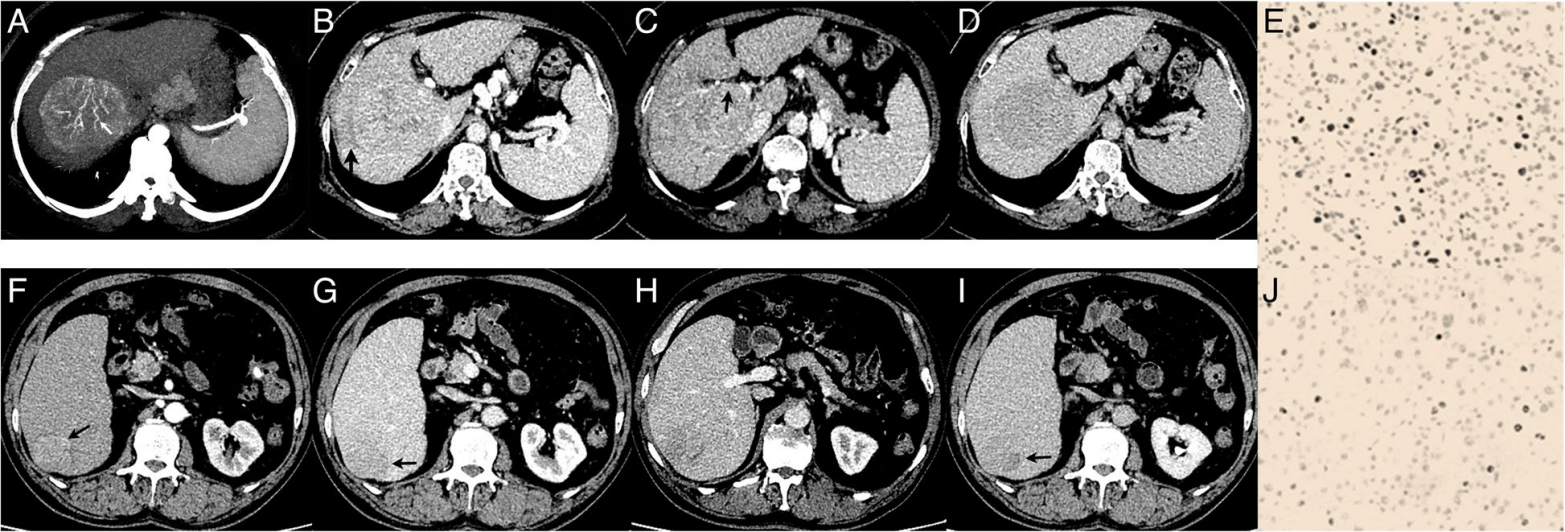
Representative images of multiphase contrast enhanced CT (CE-CT). A-E high Ki-67 HCC lession (black arrow), A, non-rim APHE and internal arteries (white arrow) on AP, B-C, hypodense halo and PVTT on PP, wash out and enhancing capsule on DP; consisting of “internal arteries” and “hypodense halos” defined as TTPVI, E, High and low Ki67 expression (70.5%), the brown regions represent positive Ki67 expression; And F-J show low Ki-67 expression HCC lesion (black arrow), F, no internal arteries on AP, G-I, no hypodense halos and PVTT on PP, non-enhancing capsule on DP, no TTPVI present. J, low Ki67 expression (8.2%); AP, arterial phase; PP, portal venous phase; DP, delayed phase; PVTT, portal vein tumor thrombus; TTPVI, two-trait predictor of venous invasion

On multivariate analyses, HCCs with high Ki-67 expression were more likely to have high serum α-fetoprotein (AFP) levels (*P* = 0.041, odds ratio [OR] 2.54, 95% CI: 1.04–6.21), non-rim APHE (*P* = 0.001, OR 15.13, 95% CI: 2.87–79.76), PVTT (*P* = 0.035, OR 3.19, 95% CI: 1.08–9.37), and TTPVI (*P* = 0.026, OR 14.04, 95% CI: 1.39–144.32) (Table 3, Fig. 3). Therefore, the CR model was developed using the four factors mentioned above and the CRR model was developed using these four factors and the radiomic signature.

**Fig. 4:**
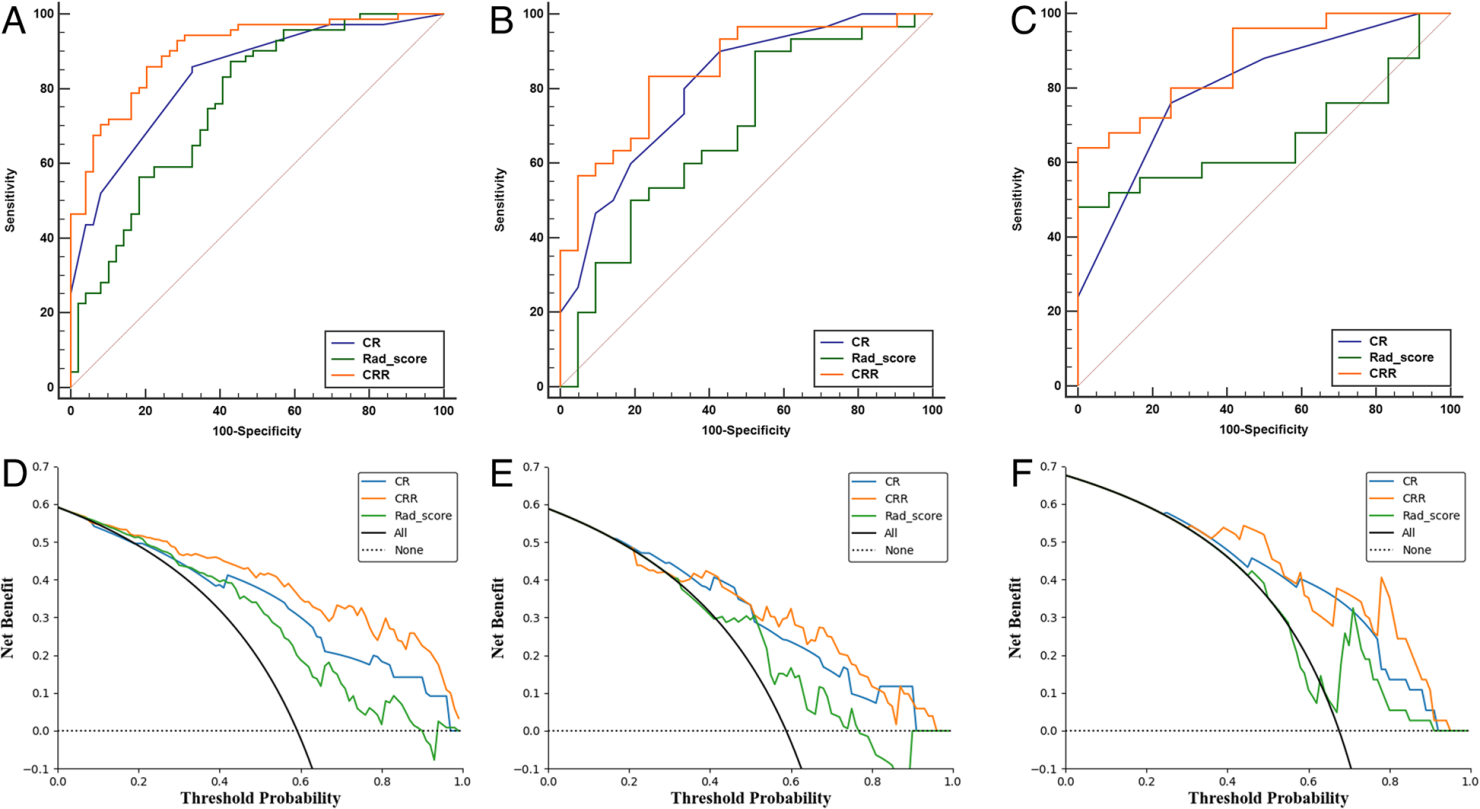
Three models predict ROC curves and DCA for KI-67-high expression. Figure A, D: training set; Figure B, E: internal test set; Figure C, F: external validation set. CR, clinico-radiologic; CRR, clinic-radiologic-radiomic

The AUC values of the CR model were 0.836 (95% CI: 0.765–0.907) in the training set, 0.805 (95% CI: 0.683–0.926) in the internal test set, and 0.805 (95% CI: 0.657–0.953) in the external validation set. The radiomic model achieved 0.762 (95% CI: 0.673–0.850) in the training set, 0.678 (95% CI: 0.536–0.839) in the internal test set, and 0.667 (95% CI: 0.495–0.839) in the external validation set. After adding the radiomic model to the CR model, the CRR model achieved 0.903 (95% CI: 0.849–0.956) in the training set, 0.848 (95% CI: 0.742–0.954) in the internal test set, and 0.877 (95% CI: 0.676–0.986) in the external validation set. The ROC curves for each model are shown in Fig. 4. After the Delong test, in the training set, there were no significant differences in the AUC values between the CR and Radiomic models, CRR and Radiomic models (*P* > 0.05), and the AUC value of the CRR model was greater than that of the CR model (*P* = 0.023). However, in the internal and external validation sets, no significant differences in the AUC values were observed between the CRR and CR models (*P* > 0.05).

In the internal test set, the sensitivity, specificity, and accuracy of the CR model were 90.0%, 71.0%, and 70.6%, respectively; for the CRR model, they were 83.0%, 76.0%, and 58.8%, respectively; and those of the radiomic model were 74.0%, 65.0%, and 47.1%, respectively. In the external test set, the sensitivity, specificity, and accuracy of the CR model were 76.0%, 75.0%, and 75.7%, respectively; for the CRR model, 64.0%, 70.0%, and 62.2%, respectively; and those of the radiomic model were 48.0%, 66.0%, and 37.8%, respectively (Table 4). After combining the rad-score and CR models, the diagnostic sensitivity, specificity, and accuracy of the CRR model did not increase.

**Table 4:** Comparison of the CR model and CRR model in three set

Models	Training set				Internal test set				External validation set			
Models	AUC value (95%CI)	Sensitivity (%)	Specificity (%)	Accuracy (%)	AUC value (95%CI)	Sensitivity (%)	Specificity (%)	Accuracy (%)	AUC value (95%CI)	Sensitivity (%)	Specificity (%)	Accuracy (%)
CR	0.836(0.765-0.907)	86	70	78.33	0.805(0.683-0.926)	90	71	70.59	0.805(0.657-0.953)	76	75	75.68
CRR	0.903(0.849-0.956)	86	79	82.5	0.848(0.742-0.954)	83	76	58.82	0.877(0.676-0.986)	64	70	62.16
Rad-score	0.762(0.673-0.850)	87	67	73.33	0.687(0.536-0.839)	74	65	47.06	0.667(0.495-0.839)	48	66	37.84

The DCA of the CRR model in the training set is an optimal decision-making strategy compared with the other two models. However, the test and external validation sets did not result in significant extra significant benefits compared with the CR model (Fig. 4).

## Discussion

In this study, we established and validated a CR model and a CRR model based on preoperative enhanced CT for the preoperative identification of high Ki-67 expression in single HCCs, and compared the predictive performance of these two models. The results indicated that the CRR model showed a higher AUC value but no statistically significant improvement over the CR model.

Radiomics has recently been introduced as a novel method for detecting Ki-67 and is considered a potential bridge that connects medical imaging and personalized medicine. The 3D volume (VOI) of the tumor can provide better morphological information and better reflect tumor heterogeneity than 2D (ROI) [13, 14]. In our study, seven radiomic features were selected that were most related to Ki-67 expression in the three phases of CE-CT. Our results showed that the AUC value of the rad-score model decreased from the training set (0.762) to the internal test set (0.687) to the external validation set (0.667), and the performance of this model was not as good as that of Wu [12], which illustrates the instability of the radiomics model despite the standardization of images and data before data analysis. The sensitivity, specificity, and accuracy of the rad-score model were unstable. In our study, the rad-score model may help to increase the prediction ability of the CR model in the training set; however, in the internal and external validation sets, there were no significant differences among the three models, indicating that radiomic features could not provide much-added value for the prediction of high Ki-67 expression HCCs, which yielded inconsistent results with other studies [5, 12]. This may be because although some published articles on the same topic of using a radiomics model based on CE-CT\Gd-EOB-DTPA-enhanced MRI to predict Ki-67 expression in HCC [5, 9, 11, 12, 15], there are many differences in details compared with our study. First, most of the studies were based on the largest on multiple HCC lesions, which vary greatly and do not explain the one-to-one correspondence between each HCC lesion and Ki**-**67 status, thus the results are unreliable. Second, radiomics research based on CT imaging has not established an external validation set to verify the stability of the radiomics model [11, 12]. In contrast, the CR model is relatively stable, both in AUC value, sensitivity, specificity, and accuracy, which is similar to the performance of the radiomics combined model of the internal test set (AUC value: 0.819) in Wu’s [12] study and is better than that in Ye’s [5] study based on MRI images. In the CRR model of the internal and external validation sets, the rad-score model had no added value for predicting Ki-67 expression preoperatively. DCA of the training set showed good clinical benefits; however, the test and external validation sets showed little clinical utility.

Most models based on radiomics methods are still in the scientific research stage and have not yet been clinically applied. This dilemma limits the social and commercial value of radiomic approaches. The generalizability of radiomics models is crucial for their clinical application. However, the reality is that most radiomics models perform well on the training data but cannot achieve stable performance in internal and external independent validation; that is, the generalization of the model is poor. There are many possible reasons, such as (1) insufficient data sample size and sample diversity, (2) poor consistency of data labeling, and (3) the special screening method was not good, and stable and universal features reflecting tumor heterogeneity could not be found. Therefore, improving the generalization of the model is an important problem that urgently needs to be solved in the field of radiomics.

Our study found that the serum AFP levels in the high and low Ki-67 groups were significantly different, consistent with previous studies [5, 16]. High serum AFP expression is correlated with more biologically aggressive properties and unfavorable tumor behaviors in HCCs [16, 17]. High serum AFP levels are more likely to be observed in highly proliferative HCCs [5]. LI-RADS major and axillary features could help in the accurate and differential diagnosis of HCC [18]. In addition, previous studies have found some specific features important in reflecting the malignancy of hepatocellular carcinoma and making treatment regimens in daily diagnostic work [19, 20]. Segal et al. first proposed TTPVI, strongly correlated with MVI and a specific HCC molecular profile related to angiogenesis, cellular proliferation, and matrix invasion [21–23]. It may also be used as a preoperative biomarker for predicting postoperative outcomes in patients with early-stage HCC [24]. Portal vein tumor thrombus (PVTT) plays a major role in the prognosis and clinical staging of HCC [25]. We assumed that these features might help improve the prognosis of HCCs with high Ki-67 expression. Thus, in our study, we analyzed both the LI-RADS features and the imaging features mentioned above. Non-rim APHE, TTPVI, and PVTT were independent predictors of high Ki**-**67 expression. As Ki-67 reflects cellular proliferation and matrix invasion, TTPVI and PVTT could help improve the prognosis of HCC with high Ki-67 expression preoperatively. After combining these three imaging features with AFP level, the CR model showed good and stable predictive performance of Ki-67, with an AUC of 0.836 (95% CI: 0.765–0.907), 0.805 (95% CI: 0.683–0.926), and 0.805 (95% CI: 0.657–0.953) in training, testing, and external validation sets, respectively, helping identify high-risk HCC groups. This is even better than the CR model based on MRI [5]. Therefore, our model based on CE-CT imaging could assist in the formulation of clinical treatment protocols, such as high-risk HCC groups that could receive advanced treatment target therapy before surgery or postoperative adjuvant transcatheter arterial chemoembolization (PA-TACE), to reduce the rate of recurrence after surgery [4, 26]. Therefore, after comprehensive analysis, the CR model was found to be the best predictive model for this study because of its stability and generalization.

This study had several limitations. First, the sample size is still small, and there may be overfitting during the establishment of the radiomics model; therefore, it is necessary to verify further the large sample and multi-center data in the future. Second, our study did not include planned CT scan imaging that could provide raw information about HCC, such as fat composition. Third, the three sets use several different models of scanning settings standby. Finally, although all images before extracting the radiomics features are resampled sampling and grayscale uniform processing, they cannot completely exclude the impact of different equipment on the radiomics features, and there is a need for future research to further solve the problem of image standardization of different institutions and different devices.

## Conclusions

As the preoperative CR model has good and stable predictive value in the preoperative prediction of Ki-67 expression in HCC, radiomics does not provide added value. Thus, there may be no need to add workforce to the addition of radiomics features in the prediction of HCC Ki-67 expression preoperatively.

### Supplementary Information

**Additional file 1:** **Supplement Figure 1.** CE-CT imaging features of HCC. A, non-rim APHE; B, non-peripheral washout and enhancing complete capsule; C, corona enhancement; D, nodule-in-nodule architecture; E, mosaic architecture; F, scar sign; G, tumor rupture; H, PVTT; I, peritumoral satellite. **Supplement Table 1.** Cohen’s kappa value of CT imaging features. **Supplement Figure 2.** The names and weights of radiomics features associated with the Ki-67 expression in training set

## Data Availability

The datasets used and analysed in the current study are available from the corresponding author on reasonable request.

## Abbreviations

HCC: Hepatocellular carcinoma
ROC: Receiver operating characteristic
LI-RADS: Liver imaging reporting and data system
AFP: Alpha-fetoprotein
AUROC: The area under the receiver operating characteristic
MVI: Microvascular invasion
TTPVI: Two-trait predictor of venous invasion
DCA: Decision curve analysis
