# Supplementary material for: Added value of CE-CT radiomics to predict high Ki-67 expression in hepatocellular carcinoma
Source: BMC Med Imaging. 2023 Sep 22;23:138. doi: 10.1186/s12880-023-01069-4 (PMC10514983; doi:10.1186/s12880-023-01069-4)
Supplement: Supplementary file 1 — Additional file 1: Supplement Figure 1. CE-CT imaging features of HCC. A, non-rim APHE; B, non-peripheral washout and enhancing complete capsule; C, corona enhancement; D, nodule-in-nodule architecture; E, mosaic architecture; F, scar sign; G, tumor rupture; H, PVTT; I, peritumoral satellite. Supplement Table 1. Cohen’s kappa value of CT imaging features. Supplement Figure 2. The names and weights of radiomics features associated with the Ki-67 expression in training set [file 12880_2023_1069_MOESM1_ESM.docx]

**Supplementary Information**

**
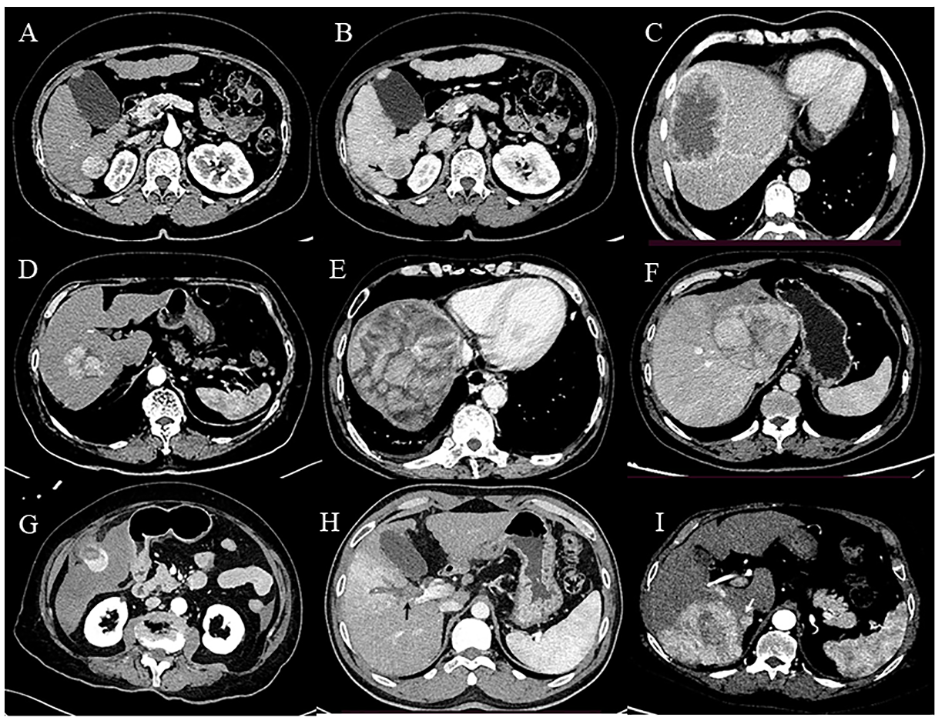
**


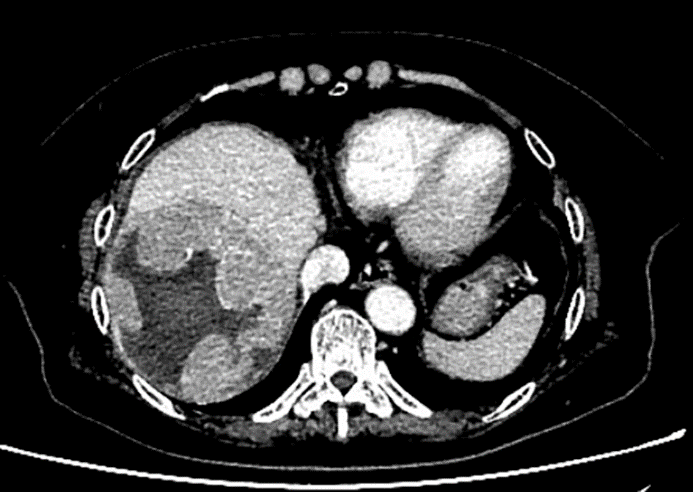
 **Supplement Figure 1** CE-CT imaging features of HCC. A, non-rim APHE; B, non-peripheral washout and enhancing complete capsule; C, corona enhancement; D, nodule-in-nodule architecture; E, mosaic architecture; F, scar sign; G, tumor rupture; H, PVTT; I, peritumoral satellite.

**Supplement Table 1** Cohen’s kappa value of CT imaging features

| CE-CT finding | Kappa value |
| --- | --- |
| Size | 0.872 |
| Non-rim APHE | 0.771 |
| Non-peripheral washout | 0.798 |
| Enhancing capsule | 0.667 |
| Completed capsule | 0.353 |
| Corona enhancement | 0.782 |
| Nodule-in-nodule architecture | 0.751 |
| Mosaic architecture | 0.731 |
| Scar sign | 0.917 |
| Tumor rupture | 0.794 |
| Intra-tumoral necrosis | 0.86 |
| PVTT | 0.861 |
| TTVPI | 0.791 |
| peritumoral satellite | 0.870 |

APHE, arterial phase hyperenhancement; PVTT, portal venous tumor; TTPVI, two-trait predictor of venous invasion

**
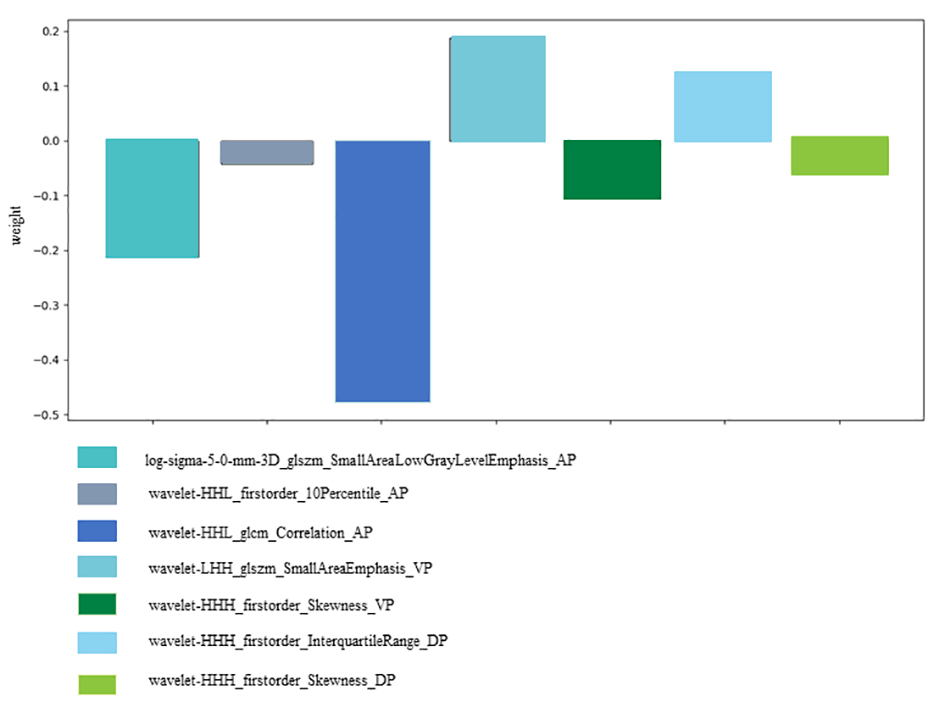
**

**Supplement Figure 2** The names and weights of radiomics features associated with the Ki-67 expression in training set.

**Supplement** **QRS-Radiomics**

1. Image protocol quality - well-documented image protocols (for example, contrast, slice thickness, energy, etc.) and/or usage of public image protocols allow reproducibility/replicability


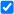
protocols are well-documented


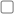
public protocol used


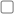
none

1. Multiple segmentations - possible actions are: segmentation by different physicians/algorithms/software, perturbing segmentations by (random) noise, segmentation at different breathing cycles. Analyse feature robustness to segmentation variabilities

yes


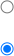

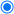


no

1. Phantom study on all scanners - detect inter-scanner differences and vendor-dependent features. Analyse feature robustness to these sources of variability

yes


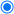

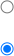


no

1. Imaging at multiple time points - collect images of individuals at additional time points. Analyse feature robustness to temporal variabilities (for example, organ movement, organ expansion/ shrinkage)

yes


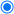

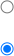


no

1. Feature reduction or adjustment for multiple testing - decreases the risk of overfitting. Overfitting is inevitable if the number of features exceeds the number of samples. Consider feature robustness when selecting features

either measure is implemented


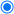

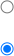


neither measure is implemented

1. Multivariable analysis with non radiomics features (for example, EGFR mutation) - is expected to provide a more holistic model. Permits correlating/inferencing between radiomics and non radiomics features

yes


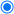

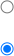


no

1. Detect and discuss biological correlates - demonstration of phenotypic differences (possibly associated with underlying gene–protein expression patterns) deepens understanding of radiomics and biology

yes


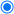

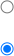


no

1. Cut-off analyses - determine risk groups by either the median, a previously published cut-off or report a continuous risk variable. Reduces the risk of reporting overly optimistic results

yes


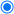

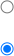


no

1. Discrimination statistics - report discrimination statistics (for example, C-statistic, ROC curve, AUC) and their statistical significance (for example, p-values, confidence intervals). One can also apply resampling method (for example, bootstrapping, cross-validation)


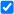
a discrimination statistic and its statistical significance are reported


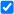
a resampling method technique is also applied


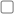
none

1. Calibration statistics - report calibration statistics (for example, Calibration-in-the-large/slope, calibration plots) and their statistical significance (for example, P-values, confidence intervals). One can also apply resampling method (for example, bootstrapping, cross-validation)


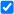
 a calibration statistic and its statistical significance are reported


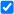
a resampling method technique is also applied


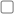
none

1. Prospective study registered in a trial database - provides the highest level of evidence supporting the clinical validity and usefulness of the radiomics biomarker

yes


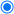

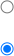


no

1. Validation - the validation is performed without retraining and without adaptation of the cut-off value, provides crucial information with regard to credible clinical performance


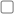
validation is based on a dataset from the same institute


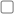
validation is based on a dataset from another institute


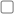

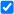
validation is based on two datasets from two distinct institutes

the study validates a previously published signature


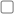
validation is based on three or more datasets from distinct institutes


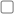
none

1. Comparison to ‘gold standard’ - assess the extent to which the model agrees with/is superior to the current ‘gold standard’ method (for example, TNM-staging for survival prediction). This comparison shows the added value of radiomics

yes


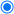

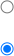


no

1. Potential clinical utility - report on the current and potential application of the model in a clinical setting (for example, decision curve analysis).

yes


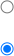

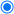


no

1. Cost-effectiveness analysis - report on the cost-effectiveness of the clinical application (for example, QALYs generated)

yes


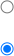

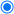


no

1. Open science and data - make code and data publicly available. Open science facilitates knowledge transfer and reproducibility of the study

scans are open source


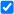

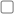


region of interest segmentations are open source


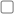
code is open source


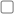
radiomics features are calculated on a set of representative ROIs and the calculated features and representative ROIs are open source

Total points: 26 (72.22%)
